# A 3D Interactive Model and Atlas of the Jaw Musculature of *Alligator mississippiensis*

**DOI:** 10.1371/journal.pone.0062806

**Published:** 2013-06-07

**Authors:** Casey M. Holliday, Henry P. Tsai, Rebecca J. Skiljan, Ian D. George, Sami Pathan

**Affiliations:** Integrative Anatomy, Department of Pathology and Anatomical Sciences, University of Missouri, Columbia, Missouri, United States of America; Raymond M. Alf Museum of Paleontology, United States of America

## Abstract

Modern imaging and dissemination methods enable morphologists to share complex, three-dimensional (3D) data in ways not previously possible. Here we present a 3D interactive model of the jaw musculature of the American Alligator (*Alligator mississippiensis*). Alligator and crocodylian jaw musculature is notoriously challenging to inspect and interpret because of the derived nature of the feeding apparatus. Using Iodine-contrast enhanced microCT imaging, a segmented model of jaw muscles, trigeminal nerve, brain and skull are presented as a cross-sectional atlas and 3D, interactive pdf of the rendered model. Modern 3D dissemination methods like this 3D Alligator hold great potential for morphologists to share anatomical information to scientists, educators, and the public in an easily downloadable format.

## Introduction

Modern technology has afforded morphologists and evolutionary biologists the ability to visualize and share the anatomy of species using a variety of imaging modalities such as serial histological reconstruction, computed tomography (CT) scanning, magnetic resonance imaging (MRI), confocal microscopy, and laser scanning among others [1], [2]. Recently, contrast techniques, such as Iodine Potassium Iodide (I_2_KI) and Phosphotungstic acid (PTA), coupled with CT imaging have enabled the visualization of soft tissues in ways not previously possible [3]–[5]. These techniques are particularly beneficial to studying musculoskeletal anatomy because the contrast techniques clarify soft tissue anatomy and organization more vividly than MRI. Segmentation analysis of these serial images then facilitates the creation of three-dimensional (3D) models that can be easily rendered into high-resolution, interactive 3D pdfs or even printed models using rapid prototyping methods [6]. Finally, the recent surge in biomechanical analyses, such as finite element modeling and multi-body dynamics analysis, has made disseminating accurate, three-dimensionally accurate complex anatomy a necessity as collaborative projects investigating the functional morphology of animal systems mature [6]–[8].

Accurate three-dimensional representations of jaw muscles, like those of *Alligator* are beneficial to many researchers including those interested in crocodylian natural history and feeding ecology, evolutionary morphology and vertebrate paleontology, and biomechanics. Interpretations from an early version of this particular model have already been applied to one investigation into the soft tissue anatomy of *Alligator* [9], [10]. But also, these small-sized, web-hosted, easily downloadable, interactive files are easily accessible by the general public, undergraduate students, and K-12 students interested in modern anatomical studies as well as charismatic extant archosaurs. Examples of shared, web-based vertebrate imaging resources can be found at Digimorph (http://www.Digimorph.org/); Aves3D (www.Aves3D.org); Paleoview (http://paleoview3d.marshall.edu/); and the 3D Alligator (web. missouri.edu/∼hollidayca/3DAnatomy/Alligator3D; http://www.oucom.ohiou.edu/dbms-witmer/3D_gator.htm). This contribution follows in the footsteps of these cutting edge, shared anatomical resources.

**Figure 1:**
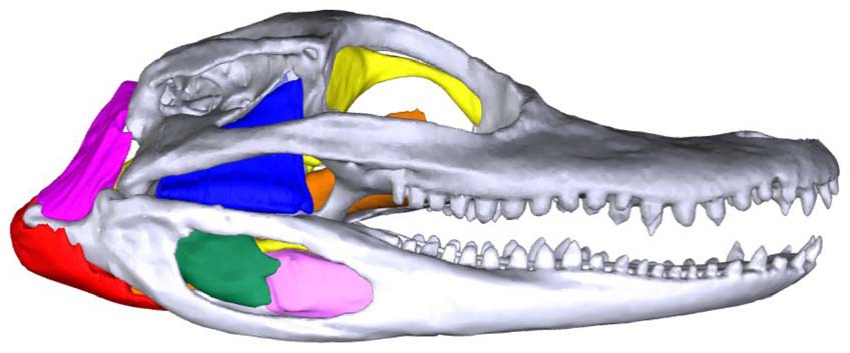
3D interactive model of the jaw musculature of *Alligator mississippiensis* as modeled from I2KI staining and CT-scanning. The 3D version of this file is available in the Supporting Information: Figure S1.

**Figure 2:**
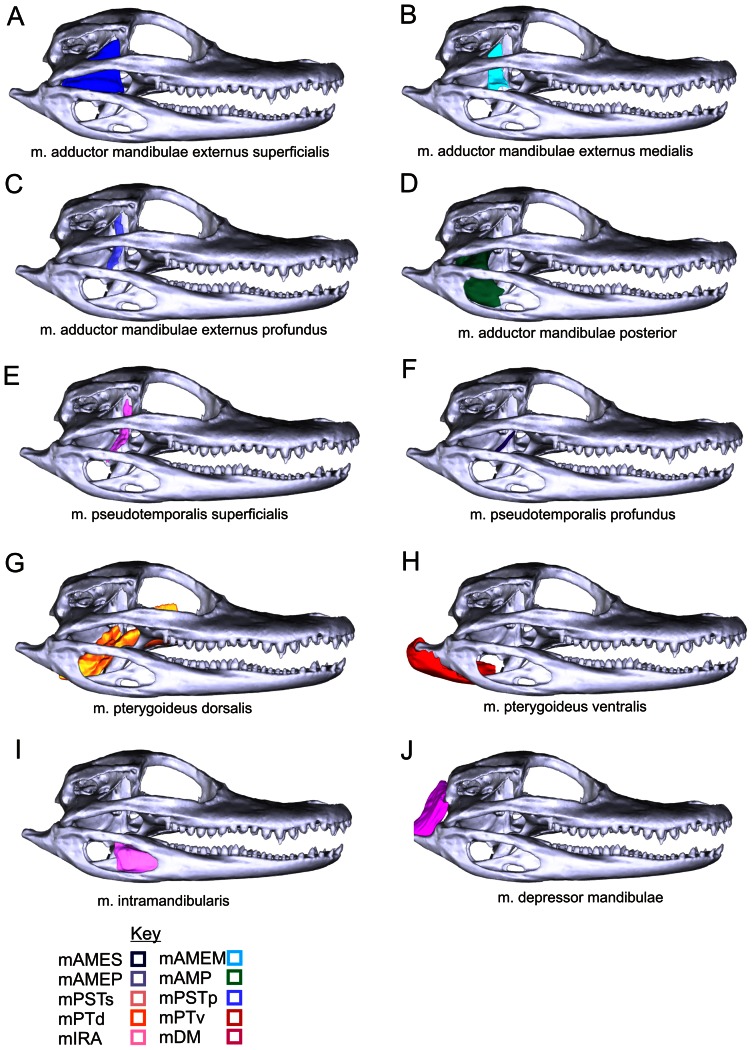
Individual jaw muscles and their attachments in *Alligator mississippiensis* as modeled from I_2_KI staining and CT-scanning. The Key pairs abbreviations of muscles with their relevant image and color in **A-J**. Abbreviations (also found in 3D PDF): mAMEM, m. adductor mandibulae externus medialis; mAMEP, m. adductor mandibulae externus profundus; mAMES, m. adductor mandibulae externus superficialis; m. adductor mandibulae posterior; mDM, m. depressor mandibulae; mIRA, m. intramandibularis; mPSTp, m. pseudotemporalis profunduis; mPSTs, m. pseudotemporalis superificialis; mPTd, m. pterygoideus dorsalis; mPTv, m. pterygoideus ventralis; mAMP.

**Figure 3:**
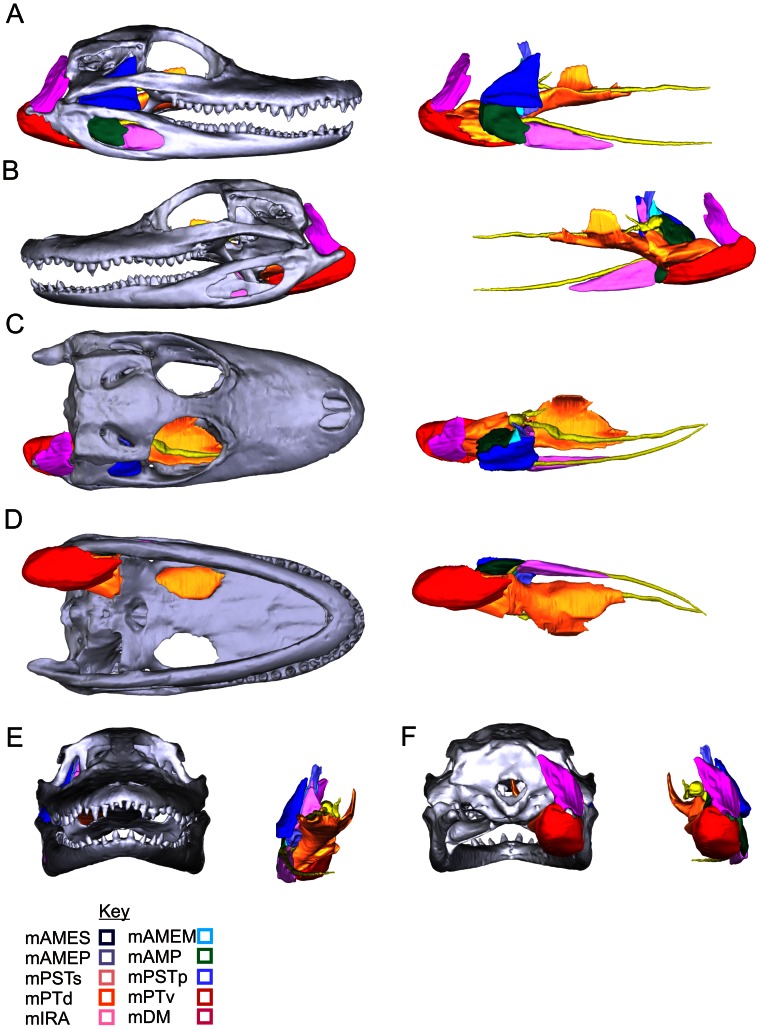
The 3D model of the jaw muscles of *Alligator mississippiensis* showing the relationship between the skull, muscles and branches of the trigeminal nerve. Left: with skull; Right: skull removed. **A**, Right lateral view; **B**, Left lateral view; **C**, Dorsal view; **D**, Ventral view; **E**, Rostral view; **F**, Caudal view.

Here we employed I_2_KI contrast staining and microCT imaging to develop the most anatomically accurate segmented three-dimensional model and interactive 3D pdf of the jaw muscles, nervous tissues, and skull of the American alligator (*Alligator mississippiensis*) (Figs. 1–3). Alligator, and crocodylian jaw musculature has been notoriously challenging to study for several reasons. First, the characteristically flat skull makes the adductor muscles, or jaw-closing muscles logistically difficult to access deep to the temporal bars, large quadrate, and robust mandible. Second, individual crocodylian muscles are generally poorly-defined compared to those of lizards, birds and mammals making their individual identification challenging [11], [12]. Third, this hypertrophied amalgam of jaw musculature has resulted in conflicting interpretations of muscle homology and evolution [13]–[16] (see Holliday and Witmer [2007] [11] for a review). In addition to the jaw musculature, we include the brain and trigeminal nerve divisions as they pass among the musculature. The relative positions of the jaw muscles and the divisions of the trigeminal nerve are classic topological characters used to interpret anatomy and homology of vertebrate jaw muscles [13]. We intend this contribution to serve as a visual atlas rather than a text-based description as the jaw muscle anatomy of *Alligator* and crocodylians have been described in numerous other papers [11]–[16].

## Results and Discussion

Measurements of the skull and muscle volumes, as well as commonly used abbreviations are found in Table 1. Abbreviations for muscles are also provided in the text following their first use.

**Table 1:** List of the materials in the 3D Alligator mode relevant l, the volumes of the segmented portions, and their abbreviations.

Material	Abbreviation	Volume (mm^3^)	Skull length (mm)	Skull Width (mm)
Cranium		3915.78	56.71	29.83
Mandibles		1360.33		
m. adductor mandibulae externus superficialis	mAMES	134.65		
m. adductor mandibulae externus medialis	mAMEM	39.03		
m. adductor mandibulae externus profundus	mAMEP	24.07		
m. pseudotemporalis superficialis	mPSTs	33.47		
m. pseudotemporalis profundus	mPSTp	2.79		
Cartilago transiliens		12.98		
m. intramandibularis	mIRA	95.32		
m. adductor mandibulae posterior	mAMP	219.01		
m. pterygoideus ventralis	mPTv	397.41		
m. pterygoideus dorsalis	mPTd	606.78		
m. depressor mandibulae	mDM	110.27		
Brain		949.83		
Trigeminal ganglion		15.05		
Ophthalmic nerve*		2.63		
Maxillary nerve		26.55		
Mandibular nerve		39.37		

* ophthalmic nerve was only partially segmented as it became impossible to see the nerve between the eye and orbit.

### Ventral view

**Figure 4:**
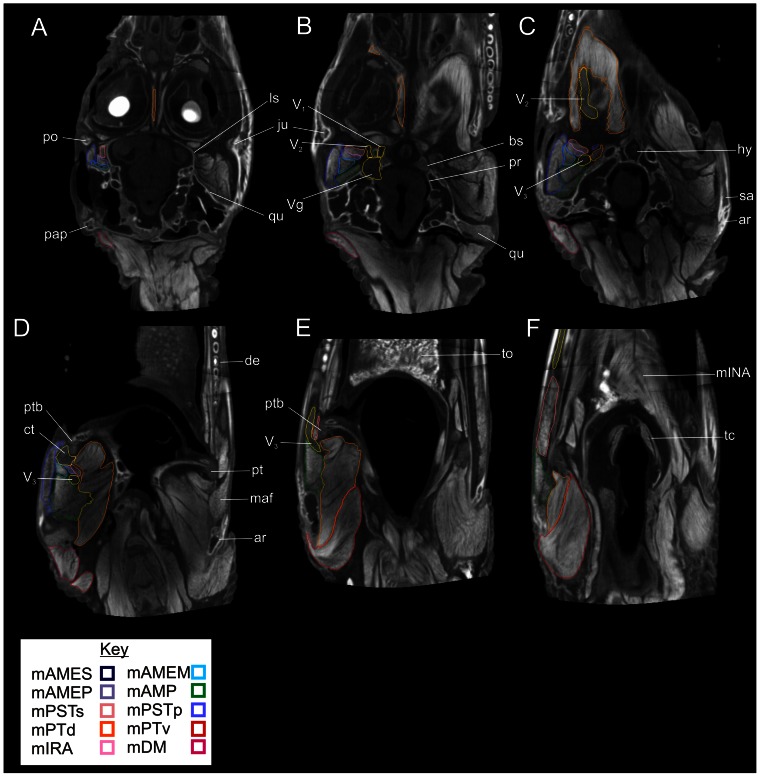
Ventral views of transverse sections through the segmented CT data showing labeled individual right jaw muscles on the left, and unlabeled left structures on the right. **A**, Slice 312/512; **B**, Slice 279/512; **C**, Slice 253/512; **D**, Slice 210/512; **E**, Slice 179/512; **F**, Slice 148/512. **Abbreviations**: **ar**, articular; **bs**, basisphenoid; **ct**, cartilago transiliens; **de**, dentary; **hy**, hypophysis; **ju**, jugal; **ls**, laterosphenoid; **maf**, mandibular fossa; **Mf**, Meckelian fossa; **mINA**, m. intermandibularis; **pap**, paroccipital process; **po**, postorbital; pr, prootic; **pt**, pterygoid; **ptb**, pterygoid buttress; **qu**, quadrate; **sa**, surangular; **tc**, thyroid cartilage; **to**, tongue; **V_1_**, ophthalmic nerve; **V_2_**, maxillary nerve; **V_3_**, mandibular nerve; **Vg**, trigeminal ganglion.

In the transverse sections (Fig. 4), beginning at the dorsalmost section, one can see the origins of many of the vertical adductor muscles including m. adductor mandibulae externus profundus(mAMEP), m. adductor externus medialis(mAMEM), m. adductor externus superficialis(mAMES), and m. pseudotemporalis superficialis(mPSTs) as they pass ventrally from the dorsotemporal fossa and lateral surfaces of the quadrate and laterosphenoid. Moving ventrally, the section passes through the trigeminal ganglion and the ophthalmic and maxillary nerves as the pass rostrally towards the face. The adductor muscles begin to expand in cross-section as they continue towards their mandibular attachments. The attachments of m. pterygoideus dorsalis(mPTd) can be seen on the cartilaginous interorbital septum. Caudally, m. depressor mandibulae passes from the occipital surface of the skull towards the retroarticular process. In the next section, the expansive origin of m. pterygoideus dorsalis is seen passing from the cavichonchal recess and is bounded dorsally by the maxillary nerve. Caudally, the mandibular nerve passes towards the Meckelian fossa of the mandible and is surrounded by musculature including m. adductor mandibulae posterior(mAMP) caudally and the pterygoid origin of m. pterygoideus dorsalis and the origin on the small m. pseudotemporalis profundus(mPTSp) medially. Further ventrally, the mandibular nerve begins to curve rostrally towards the corner of the mouth and the expansive pterygoid origins of m pterygoideus ventralis(mPTv)are visible. In this section, near the corner of the mouth, the large sesamoid cartilago transiliens can be seen linking mm. adductor mandibulae externus profundus, pseudotemporalis superficialis, and pterygoideus dorsalis dorsally with m. intramandibularis(mIRA) ventrally. The next section passes ventrally through the dorsal part of the mandible. The insertions of m. adductor mandibulae posterior into the mandibular fossa and m. pterygoideus dorsalis into the medial surface of the articular are visible. The mandibular nerve is seen passing laterally to m. intramandibular within the rostral portion of the Meckelian fossa. Caudally, m. pterygoideus ventralis is visible passing around the retroarticular process. Finally, the ventralmost section shows the mandibular nerve passing rostrally towards the chin. The bellies of mm. intramandibularis, adductor mandibular posterior and pterygoideus dorsalis and ventralis are visible as they attach to the mandible.

### Rostral view

**Figure 5:**
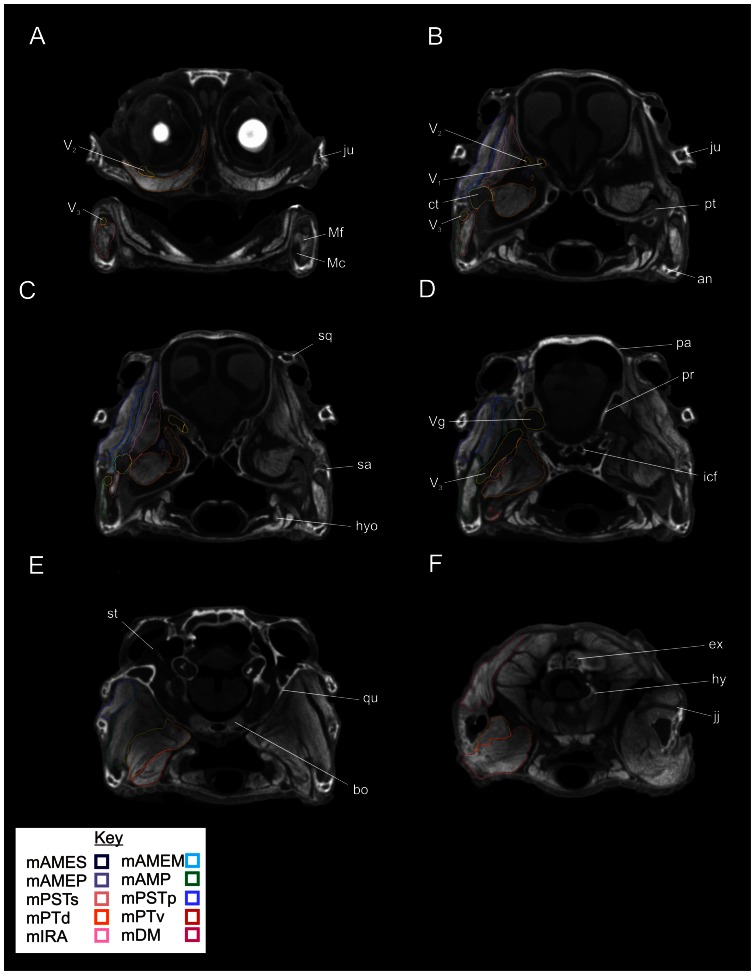
Rostral views of axial sections through the segmented CT data showing labeled individual right jaw muscles on the left, and unlabeled left structures on the right. **A**, Slice 391/911; **B**, Slice 316/911; **C**, Slice 305/911; **D**, Slice 283/911; **E**, Slice 240/911; **F**, Slice 154/911. **Abbreviations**: **an**, angular; **bo**, basioccipital; **ct**, cartilage transiliens; **ex**, exoccipital; **hyo**, hyoid; **icf**, internal carotid foramen; **jj**, jaw joint; **ju**, jugal; **Mc**, Meckel's cartilage; **Mf**, Meckelian fossa; **naa**, neural arch of atlas; **pa**, parietal; **pr**, prootic; **pt**, pterygoid; **qu**, quadrate; **sa**, surangular; **sq**, squamosal; **st**, stapes; **V_1_**, ophthalmic nerve; **V_2_**, maxillary nerve; **V_3_**, mandibular nerve; **Vg**, trigeminal ganglion.

In the axial sections (Fig. 5), beginning at the rostralmost section, m. pterygoideus dorsalis and the maxillary nerve are visible passing ventral to the orbit. Ventrally, the mandibular nerve is visible passing dorsal to m. intramandibularis within the Meckelian fossa. In the next section caudally, many of the adductor muscles are visible as they pass from the lateral wall of the braincase towards the mandible. The cartilago transiliens is visible linking bellies of m. adductor mandiblue externus and m. pseudotemporalis superficialis with m. intramandibularis as it wraps laterally around the pterygoid buttress. Medially, the ophthalmic and maxillary nerves are separated by the laterosphenoid lateral bridge. In the next section, the laminar pattern of the adductor muscles is easily appreciated with m. adductor mandibulae externus superficialis located laterally followed medially by the other vertical adductors and finally m. pseudotemporalis profundus and m. pterygoideus dorsalis. The next section occurs in a plane through the trigeminal ganglion and the mandibular nerve. Ventral to the nerve are m. pterygoideus dorsalis and the beginnings of m. pterygoideus ventralis as it comes off of the caudal edge of the pterygoid buttress. The next section caudally shows m. adductor mandibulae externus superficialis attaching to the dorsal surface of the surangular, m. adductor mandibulae posterior passing from the surface of the quadrate to the mandibular fossa and then mm. pterygoideus dorsalis and ventralis passing caudally towards the caudal end of the mandible. Finally, the caudalmost section passes through the neck and retroarticular process. The majority of m. depressor mandibulae is visible as it passes from the caudal surface of the paroccipital process to the dorsal surface of the retroarticular process. Ventrally the fibers of m. pterygoideus ventralis are visible passing underneath the mandible.

### Lateral view

**Figure 6:**
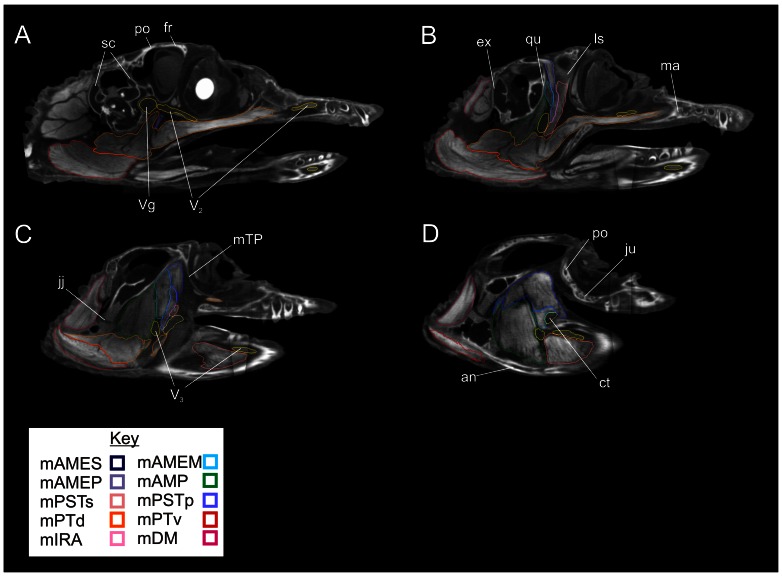
Lateral views of parasagittal sections through the segmented CT data showing labeled individual right jaw muscles. **A**, Slice 183/512; **B**, Slice 155/512; **C**, Slice 133/512; **D**, Slice 119/512. **Abbreviations**: **an**, angular; **ct**, cartilago transiliens; **ex**, exoccipital; **ju**, jugal; **ls**, laterosphenoid; **ma**, maxilla; **mTP**, m. tensor periorbitae; **po**, postorbital; **qu**, quadrate; **sc**, semicircular canals; **V_2_**, maxillary nerve; **V_3_**, mandibular nerve; **Vg**, trigeminal ganglion.

In the parasagittal sections (Fig. 6), beginning at the medialmost section, the trigeminal ganglion, maxillary nerve and majority of the origin of m. pterygoideus dorsalis are visible. In the next section lateral, the mandibular nerve passes ventrolaterally towards the mandible and is surrounded by numerous adductor muscles. The origin of m. adductor mandibulae externus profundus is visible in the dorsotemporal fossa as is the origin of m. pseudotemporalis superficialis on the lateral surface of the laterosphenoid. The large belly of m. pterygoideus ventralis is visible originating from the caudal edge of the pterygoid buttress. The cartilago transiliens is visible on the dorsolateral edge of the pterygoid buttress. The mandibular nerve and m. intramandibularis are visible within the Meckelian fossa. Finally, the lateralmost parasagittal section shows m. adductor mandibulae posterior filling the mandibular fossa medial to the mandibular fenestra. Caudally, m. depressor mandibulae and m. pterygoideus ventralis respectively attach to the dorsal and ventral portions of the retroarticular process. Dorsally, the obliquely vertical fibers of m. adductor mandibulae externus superficialis and medialis are visible passing towards the surangular.

## Conclusions

Alligator jaw musculature, like that of other vertebrates, is three-dimensionally complex and difficult to illustrate in two-dimensional media. Here we provided the first volumetric model of *Alligator*, and crocodylian musculature based on raw imaging data. This downloadable, 3D model of *Alligator* jaw muscles, skull and nervous tissues shows the potential the next generation of anatomical tools have for morphologists, biologists, and the general public.

## Materials and Methods

One 12-month-old fresh-frozen cadaveric *Alligator mississippiensis* was obtained from Rockefeller State Refuge, Grand Chenier, Louisiana, US and accessioned into the University of Missouri Vertebrate Collections as MU AL031. The head was removed, fixed in 10% neutral buffered formalin and stored in 70% ethanol. The head was then immersed in 10% Iodine Potassium Iodide (i.e., I_2_KI; Lugol's Iodine) for 5 weeks with 2 intervening CT scans to check on diffusion success. The final scan was conducted on a Siemens Inveon MicroCT scanner using 80 kV, 500 mA, and slice thickness of 83 microns at the University of Missouri Biomolecular Imaging Center. Scan data were imported as DICOM files into Amira v 5.2 (Visage Imaging) for segmentation. Anatomical structures were segmented manually using both magic wand and paintbrush tools by the authors. A defect in the scanner resulted in an obvious stitching artifact along the z-axis of the scan visible passing through the junction of the face and braincase. This did not significantly impact the interpretation of the data. Individual segmented structures were saved as STL files, smoothed and cleaned in Geomagic 12, and then imported into Adobe Acrobat 3D v.8 Toolkit, further reduced, and organized into a 3D model. This file was then imported into Adobe 8 3D and organized into its final format. Screen captures of specific slices of the segmented model were taken from Amira to show the outlines of muscles and soft tissues.

### Ethics Statement

A cadaveric alligator was provided by Ruth Elsey, Rockefeller Wildlife Refuge, Grand Chenier, Louisiana under their permits.

## Supporting Information

Figure S1 **3D interactive model of the jaw musculature of *Alligator mississippiensis* as modeled from I2KI staining and CT-scanning.**
(PDF)
