# Supplementary figures and images for: A 3D Interactive Model and Atlas of the Jaw Musculature of Alligator mississippiensis
Source: PLoS One. 2013 Jun 7;8(6):e62806. doi: 10.1371/journal.pone.0062806 (PMC3676386; doi:10.1371/journal.pone.0062806)

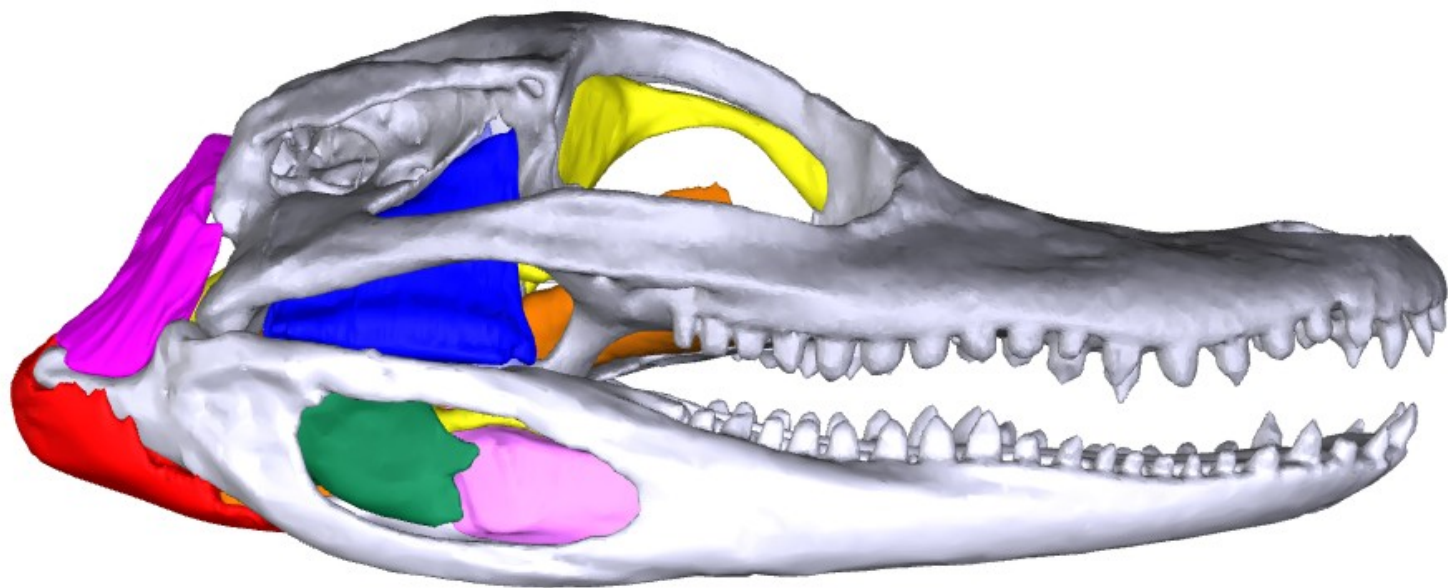

Supplement: Figure S1 — 3D interactive model of the jaw musculature of Alligator mississippiensis as modeled from I2KI staining and CT-scanning. (PDF) [file pone.0062806.s001.pdf]
